# MLPA is a practical and complementary alternative to CMA for diagnostic testing in patients with autism spectrum disorders and identifying new candidate CNVs associated with autism

**DOI:** 10.7717/peerj.6183

**Published:** 2019-01-09

**Authors:** Pavlina Capkova, Josef Srovnal, Zuzana Capkova, Katerina Staffova, Vera Becvarova, Marie Trkova, Katerina Adamova, Alena Santava, Vaclava Curtisova, Marian Hajduch, Martin Prochazka

**Affiliations:** 1 Department of Medical Genetics, University Hospital Olomouc, Olomouc, Czech Republic; 2 Institute of Molecular and Translational Medicine, Faculty of Medicine and Dentistry, Palacky University Olomouc, Olomouc, Czech Republic; 3 Gennet, s.r.o., Prague, Czech Republic

**Keywords:** Autism spectrum disorders, Copy number variants, Multiplex ligation-dependent probe amplification, *DOCK8*, Chromosomal microarray analysis

## Abstract

**Background:**

Autism spectrum disorder (ASD) is a complex heterogeneous developmental disease with a significant genetic background that is frequently caused by rare copy number variants (CNVs). Microarray-based whole-genome approaches for CNV detection are widely accepted. However, the clinical significance of most CNV is poorly understood, so results obtained using such methods are sometimes ambiguous. We therefore evaluated a targeted approach based on multiplex ligation-dependent probe amplification (MLPA) using selected probemixes to detect clinically relevant variants for diagnostic testing of ASD patients. We compare the reliability and efficiency of this test to those of chromosomal microarray analysis (CMA) and other tests available to our laboratory. In addition, we identify new candidate genes for ASD identified in a cohort of ASD-diagnosed patients.

**Method:**

We describe the use of MLPA, CMA, and karyotyping to detect CNV in 92 ASD patients and evaluate their clinical significance.

**Result:**

Pathogenic and likely pathogenic mutations were identified by CMA in eight (8.07% of the studied cohort) and 12 (13.04%) ASD patients, respectively, and in eight (8.07%) and four (4.35%) patients, respectively, by MLPA. The detected mutations include the 22q13.3 deletion, which was attributed to ring chromosome 22 formation based on karyotyping. CMA revealed a total of 91 rare CNV in 55 patients: eight pathogenic, 15 designated variants of unknown significance (VOUS)—likely pathogenic, 10 VOUS—uncertain, and 58 VOUS—likely benign or benign. MLPA revealed 18 CNV in 18 individuals: eight pathogenic, four designated as VOUS—likely pathogenic, and six designated as VOUS—likely benign/benign. Rare CNVs were detected in 17 (58.62%) out of 29 females and 38 (60.32%) out of 63 males in the cohort. Two genes, *DOCK8* and *PARK2*, were found to be overlapped by CNV designated pathogenic, VOUS—likely pathogenic, or VOUS—uncertain in multiple patients. Moreover, the studied ASD cohort exhibited significant (*p* < 0.05) enrichment of duplications encompassing *DOCK8*.

**Conclusion:**

Multiplex ligation-dependent probe amplification and CMA yielded concordant results for 12 patients bearing CNV designated pathogenic or VOUS—likely pathogenic. Unambiguous diagnoses were achieved for eight patients (corresponding to 8.7% of the total studied population) by both MLPA and CMA, for one (1.09%) patient by karyotyping, and for one (1.09%) patient by FRAXA testing. MLPA and CMA thus achieved identical reliability with respect to clinically relevant findings. As such, MLPA could be useful as a fast and inexpensive test in patients with syndromic autism. The detection rate of potentially pathogenic variants (VOUS—likely pathogenic) achieved by CMA was higher than that for MLPA (13.04% vs. 4.35%). However, there was no corresponding difference in the rate of unambiguous diagnoses of ASD patients. In addition, the results obtained suggest that *DOCK8* may play a role in the etiology of ASD.

## Introduction

Autistic spectrum disorders (ASDs) are complex heterogeneous developmental diseases with a significant genetic background and include three closely related diagnoses: autistic disorder, Asperger syndrome, and pervasive developmental disorder-not otherwise specified (PDD-NOS). They are characterized by simultaneous deficits in three domains of behavior: reciprocal social interaction, communication, and stereotyped and restricted behaviors. Their estimated prevalence in the population is 1:68, with males being predominantly affected (McCarthy, 2014; De La Torre-Ubieta et al., 2016; Schaefer, 2016; Schaefer & Mendelsohn, 2013; Miller et al., 2010). ASD occurs frequently with the following comorbidities: a motor deficit, sleep abnormalities, gastrointestinal disturbances, epilepsy, and intellectual disability (ID) (De La Torre-Ubieta et al., 2016; Schaefer, 2016). These comorbidities can also overlap with phenotypes observed in other neuropsychiatric disorders.

Submicroscopic copy number variants (CNVs) may have a causal or susceptibility-related role in the heritability of ASD. However, the causality and/or pathogenicity of these CNV is largely unknown due to their variable expression and incomplete penetrance, which can result in a spectrum of phenotypes ranging from asymptomatic to ID/developmental delay (DD)/ASD. Many studies have sought to identify new candidate genes for ASD but fewer have sought to clarify their clinical significance for patients and their families. Chromosomal microarray analyses (CMAs) were recently identified as a first-tier method for testing in patients with ID/DD/ASD (Schaefer, 2016; Schaefer & Mendelsohn, 2013; Miller et al., 2010; South et al., 2013). However, little is currently known about the clinical significance of most CNV, which can hamper the interpretation of test results and complicate genetic counseling. Unlike CMA, multiplex ligation-dependent probe amplification (MLPA) is fast and provides easily interpreted results. It can therefore serve as a clinically effective targeted test to detect recurrent CNV associated with ASD, and is currently used as a preliminary test to exclude recurrent pathogenic CNV in our department.

This study compares the efficiency and reliability of MLPA and CMA in diagnostic testing of ASD patients. A secondary objective was to identify new candidate genes for ASD. We present results obtained by using a combination of MLPA and CMA to analyze CNV in a cohort of 92 children diagnosed with ASD.

## Materials and Methods

### Participants

The study involved 92 individuals of Caucasian ethnicity—63 males and 29 females—with autism (54), PDD-NOS (35), and Asperger syndrome (3), predominantly from simplex families (89%). These patients were referred to genetic counseling solely on the basis of an ASD, PDD-NOS, or Asperger syndrome diagnosis, or based on some level of neurodevelopmental impairment together with ASD or PDD-NOS. The size of the study population is approximately 1/5th of the number of child patients who undergo genetic testing in our department each year. Patients with known monogenic syndromes (e.g., familial cases and clear syndromic cases) associated with ASD (tuberous sclerosis, neurofibromatosis, etc.) were excluded. Peripheral blood samples were collected after genetic counseling in the Department of Medical Genetics at the University Hospital in Olomouc, Czech Republic, during the years 2012–2016. For 17 patients, the DNA of both parents was obtained to verify the origin of the detected variants. The study was approved by the Institutional Review Board of the University Hospital and Faculty of Medicine and Dentistry, Palacky University, Olomouc (IRB number 96/17). All procedures were conducted in accordance with the Declaration of Helsinki. Written informed consent for the use of personal particulars and genetic information for research purposes was collected from the patients’ parents or guardians during genetic counseling. The cohort’s mean age at evaluation was 5.0 ± 2.9 years.

The patients underwent rigorous examinations by pediatricians, neurologists, psychiatrists, and geneticists, including metabolic tests and brain imaging. Individuals were diagnosed with ASD by clinicians after performing the Autism Diagnostic Interview-Revised and Autism Diagnosis Observation Schedule. The subjects with pervasive developmental disorders and varying levels of impairment were diagnosed with broad-spectrum disorder, which includes conditions such as PDD-NOS and Asperger syndrome. Phenotype descriptions for patients with pathogenic/variants of unknown significance (VOUS)—likely pathogenic and VOUS—uncertain findings are presented in the Supplemental File. The frequency of CNV overlapping the *DOCK8* gene in the healthy population was determined by MLPA analysis (using the SALSA MLPA P385-A2 DOCK8 probemix) of 40 male and 40 female control individuals exhibiting no related health conditions.

### Study design

The study used a retrospective observational design.

## Methods

Systematic screening for pathogenic mutations was performed by karyotyping, fragile X syndrome testing, screening for metabolic disorders, targeted MLPA testing, and CMA. Cytogenetic analysis was performed using cultured lymphocytes by conventional G-banding with a resolution of 550 bphs. DNA was isolated from peripheral blood by the saline method. DNA samples were tested for the *FMR1* mutation by PCR using fluorescently labeled primers as described previously (Zhou, 2006). MLPA tests were performed with SALSA^®^ MLPA^®^ sets P070-B3 and P036-E3 (the Subtelomeres Mix 1 and Mix 2B probemixes), P245-B1 and P297-B2/C1 (the Microdeletions 1A and 2 probemixes), P343-C3 (the Autism1 probemix), and P106-C1 (the Mental retardation X-linked probemix) in accordance with the manufacturer’s protocol. The Coffalyser program was used for CNV calling (MRC-Holland, Amsterdam, Netherlands). PCR products were identified and quantified by capillary electrophoresis on an ABI 3130 genetic analyzer, using the Gene Mapper software from Applied Biosystems, Foster City, CA, USA. Other MLPA probemixes (P051/P052-D1 and P385-A2) were used to verify CMA findings relating to *PARK2* and *DOCK8*.

Chromosomal microarray analyses were performed using a Cytoscan HD (Affymetrix, Santa Clara, CA, USA) or CytoSNP-12 (Illumina, San Diego, CA, USA) instrument according to the manufacturer’s protocol. The data discussed in this publication have been deposited in NCBI’s Gene Expression Omnibus database (Edgar, Domrachev & Lash, 2002) and are accessible using GEO Series accession number GSE114870. The programs CHAS v. 1.2.2 and Illumina KaryoStudio 1.3 from Genome Studio V2011.1 were used for CNV calling. The observed CNVs were compared to CNV recorded in the DGV to assess their frequency in the population. CNV encompassing coding regions with frequencies of <1% in the population were considered rare variants. The clinical significance of individual CNV was evaluated according to the ACMG Standard and Guidelines (Kearney et al., 2011). Where possible, parental samples were collected for patients exhibiting pathogenic CNV and VOUS to determine the CNV’s origin. Detected CNVs were systematically compared to CNV recorded in curated databases (ISCA, 2013; Firth et al., 2009, SFARI, and DGV) to determine their clinical significance. Fisher’s exact test was used to calculate the statistical significance of the frequency of *DOCK8* duplication in the ASD population (www.socscistatistics.com/tests/fisher/Default2.aspx). As a control, we used data from population sequencing studies held by the 1000 Genomes Consortium (Mills et al., 2011).

## Results

Karyotyping revealed chromosomal aberrations in three (3.26%) patients from the cohort: one pathogenic r(22)(q13.3)(1.08%) and two likely benign t(10;11)(q26;p13)pat, inv(Y)(p11.2q11.23) (Table 1). The *FMR1* mutation was identified in one (1.09%) individual. In total, MLPA revealed 18 CNVs in 18 (19.57%) individuals: eight (44.44%) pathogenic (including a terminal deletion in ring chromosome 22), four (22.22%) designated as VOUS—likely pathogenic and confirmed by CMA, and six (33.33%) designated as VOUS—likely benign/benign. All but three of these detected variants were confirmed by CMA. The variants not confirmed by CMA were small deletions (*MAPK3*—pat inherit., *SNRPN*—mat inherit., *FRG1*—pat inherit.) identified using one probe; these deletions probably correspond to SNV that were inherited from healthy parents and lie in the probe’s hybridization region. This phenomenon has been described previously and represents an inherent limitation of MLPA (Cai et al., 2008).

**Table 1:** The number of ASD patients with detected rare CNV for different method.

Method	Pathogenic CNV	%	VOUS likely pathogenic	%	VOUS uncertain	%	VOUS likely benign	%	Patients with rare variants totally	%	Negative
**karyo**	1	1.09	0	0	0	0	2	2.17	2^a^ + 1*	3.26	89
**FMR1**	1	1.09	–	–	–	–	–	–	1	1.09	91
**MLPA**	7 + 1*	8.69	4	4.35	0	0	6	6.52	18***	19.57	74
**CMA**	7 + 1*	8.69	8 + 4**	13.04	6	6.52	26 + 3**	31.52	55****	59.78	37
	9	9.78	12	13.04	6	6.52	34	36.96	61	66.3	31

**Notes:**

* Detected by karyotyping, MLPA and CMA: r(22)(q13.3).

** Detected by MLPA and CMA.

*** 15 confirmed by CMA, three not confirmed.

**** CNVs of coding region with frequency <1% in population.

a t(10;11)(q26;p13)pat; inv(Y)(p11.2q11.23).

Chromosomal microarray analysis identified 91 rare CNVs (60 duplications and 31 deletions) that contained coding regions and had MAF values of <1% in 55 (59.78%) patients. Among these were eight (8.79%) pathogenic CNVs, 15 (16.48%) CNVs designated as VOUS—likely pathogenic, 10 (10.99%) designated as VOUS—uncertain, and 58 (63.73%) designated as VOUS—likely benign or benign (Table 2). The percentages of rare CNV in the males (60.32%) and females (58.62%) of our cohort were similar.

**Table 2:** The list of detected CNV.

A. Pathogenic CNV								
Patient ID	Band	CNV status	Region GRCh37/hg19	Length (kb)	Inheritance	Gender	Method of detection	Syndrome (phenotype MIM number)
D980/11	1q21.1-q21.2	Gain	146476526–147825662	1,349	De novo	F	MLPA (P297), CMA	dup 1q21.1 (612475)
D1277/08	7q11.22	Loss	72701018–74143060	1,442	De novo	M	MLPA (P245), CMA	WBS (194050)
D1522/16	15q11.2-q13.1	Gain	20737094–31293264	10,556	NA (maternal excluded)	M	MLPA (P297, P343, P070, P036, P245), CMA	dup 15q11q13 (608636)
D731/15	16p11.2	Loss	29432212–30190029	758	Paternal	M	MLPA (P297, P343), CMA	del 16p11.2 (611913)
D767/14	16p11.2	Gain	29600878–30177240	576	Paternal	M	MLPA (P297, P343), CMA	dup 16p11.2 (614671)
D1981/12	22q11.21	Loss	20733667–21460220	727	NA	M	MLPA (P245), CMA	del 22q11 (192430)
1764/16	22q13.31-q13.33	Loss	47349588–51197838	3,848	De novo	F	MLPA (P070, P036, P343, P245), karyo r(22)(q13.3), CMA	Phelan–McDermid sy (606230)
770/16	Xp21.1-q21.2	Loss	31518523–31948537	430	NA	M	MLPA (P245), CMA	BMD (300376)

B. VOUS—likely pathogenic CNV								
Patient ID	Band	CNV status	Region GRCh37/hg19	Length (kb)	Inheritance	Gender	Method of detection	Gene of interest
D1377/15***	1q42.2	Gain	231712603–231816159	104	NA	M	CMA	DISC1
D1377/15***	10q21.3	Loss	68312378–68445989	134		M	CMA	CTNNA3
D1377/15***	8p23.2	Loss	3879391–4100961	222	NA	M	CMA	CSMD1
D357/12	2p16.3	Loss	50943528–51041472	98	Maternal	M	CMA	NRXN1
D1320/14	6q26	Loss	162631070–162982289	351	Paternal	M	CMA	PARK2
D1123/15	6q26	Gain	162716322–162912832	197	NA	M	CMA	PARK2
D714/10	9p24.3	Gain	1–271132	271	NA	M	CMA	DOCK8
D684/13	9p24.3	Gain	203861–398865	195	NA	M	CMA, MLPA (P070)	DOCK8
D1748/15	9p24.3	Gain	271533–440683	169	Paternal	M	CMA, MLPA (P070)	DOCK8
D840/15	16p13.3	Loss	7212403–7459707	247	NA	M	CMA	RBFOX1
D1327/13	20p12.1	Loss	14806577–15042599	236	NA	F	CMA	MACROD2
D781/16	Xp22.11p21.3	Gain	23794728–28429500	4,635	De novo	F	MLPA (P106-MRX), CMA	APOO, POLA1, ARX*
D1094/16**	Xp22.33	Gain	1755742–1821317	66	NA	M	CMA	ASMT*
D1194/15	Xp11.4	Gain	38486618–38634614	148	NA	F	MLPA (P106-MRX), CMA	TSPAN7
D1190/16***	Xq27.3	Gain	138614319–143156110	4 541	NA	M	CMA	SOX3, F9, ATP11C, CDR1, LDOC1*

C. VOUS—uncertain CNV								
Patient ID	Band	CNV status	Region GRCh37/hg19	Length (kb)	Inheritance	Gender	Method of detection	Gene of interest
D1474/15	2p12	Loss	80271129–80377518	106	NA	F	CMA	CTNNA2
D538/16	2q13	Loss	110874326–111365996	492	Maternal	F	CMA	NPHP1
D1471/14	6q22.33	Loss	128633613–128811348	178	NA	M	CMA	PTPRK*
D970/14	9q33	Gain	124034190–12444190	408	NA		CMA	GSN, STOM, DAB2IP, EPB72*
D1190/16***	11q14.3-q21	Gain	90076743–93118662	3,040	NA	M	CMA	MTNR1B (melatonin receptor), FAT3, SLC36A4*
D1190/16***	5q32.2	Gain	174151663–174157517	5	NA	M	CMA	MSX2
D1377/15***	16q11.2	Gain	46600773–46830637	230	NA	M	CMA	MYLK3, ORC6, SHCBP1, VPS35
D1116/12	16p12.2	Gain	21591157–21951415	360	NA (maternal excluded)	M	CMA	OTOA, METTL9, IGSF6
D2121/13	17q21.32	Gain	42212006–42542693	331	NA	F	CMA	GRN, ITGA2B, SLC4A1, ASB16, GPATCH8, RUNDC3A, SLC25A39, UBTF, SHC1P2*
D1094/16**	Xp11.22	Gain	53444924–53459515	15	NA	M	CMA	SMC1A, HSD17B10

**Notes:**

* Not in DGV.

** Patient with FMR1 mutation.

*** Patients with multiple CNV.

Multiplex ligation-dependent probe amplification and CMA yielded concordant results for all eight patients (8.7% of the study cohort) exhibiting pathogenic mutations. CNV designated VOUS—likely pathogenic were identified as primary CMA findings in 12 individuals (13.04% of the cohort), but only four (4.35%) of these were also discovered by MLPA (Table 1). The rate of detection for CNV designated as VOUS—likely pathogenic when using CMA was thus 8.69 percentage points higher than that achieved using MLPA. This difference was significant (*p* = 0.039). These CNVs represent potentially harmful mutations but there is currently insufficient evidence to classify them as being causal of the patient’s disorder. The rate of pathogenic variant detection using MLPA and CMA was 7.61 percentage points higher than that achieved by karyotyping alone in the cohort of ASD patients. A terminal deletion of chromosome 22 affecting the gene *SHANK3* was detected by all methods in the patient with ring chromosome 22. However, without karyotyping, this deletion’s mechanism of occurrence would not have been determined. The duplication 15q11.2q13.1 (patient 1522/16) was identified as a chromosome heteromorphism during cytogenetic assessment, but both MLPA and CMA revealed the duplication.

Eight recurrent CNVs known to be associated with ASD were found in our cohort—deletions 7q11.2 (Williams–Beuren syndrome; WBS), 22q11.2 (Velocardiofacial Syndrome; VCFS), 22q13 (Phelan–McDermid syndrome), 16p11.2, and Xp21.2-p21.1 (Becker muscular dystrophy; BMD); and duplications 1q21.1, 16p11.2, and 15q11-q13 (Table 2A). Several CNV encompassed genes reported to be important in the etiology of autism or schizophrenia (*APOO*, *ARX*, *TSPAN7*, *NRXN1*, *CSMD1*, *CTNNA3*, *RBFOX1*, *MACROD2*, *ASMT*, *DISC1*, *PARK2*, *DOCK8*); these CNVs were designated “likely pathogenic” because they are listed in curated databases (SFARI) or have repeatedly been identified as being involved in the etiology of autism or ID (Table 2B).

**Figure 1:**
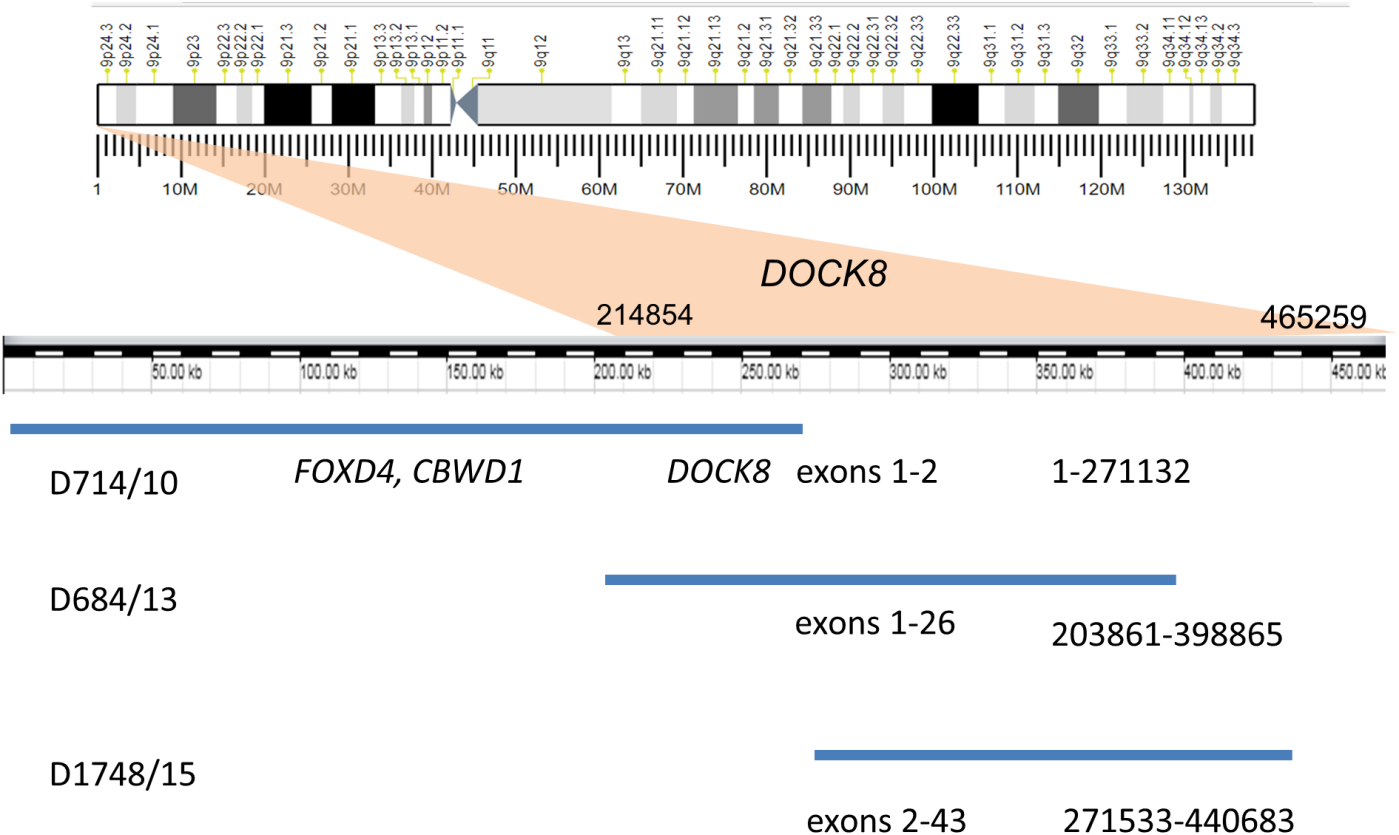
Duplication of the gene DOCK8 in three unrelated patients.

Recurrent duplications (9p24.3) overlapping the *DOCK8* gene (specifically, spanning exons 1–2, 2–43, and 1–26) were detected in three unrelated patients by MLPA and CMA (Fig. 1). All of these duplications are currently recorded as variants of unknown clinical significance. A similar MLPA analysis of a control cohort of 80 healthy individuals revealed no individuals with CNV encompassing this gene. Moreover, *DOCK8* gain variants were found in only 12 of the 2,504 healthy individuals whose genetic data were published by the 1,000 Genomes Consortium (Mills et al., 2011). *DOCK8* gain variants are thus significantly enriched in the ASD cohort relative to the population as a whole (*p* < 0.05).

We also analyzed the phenotypes of patients with similar duplications encompassing the gene *DOCK8* that have been reported in the literature and the ISCA and DECIPHER databases (Table 3). The analyzed cases exhibited similar phenotypes involving ASD, DD, speech delay seizures, dysmorphic features and behavioral abnormalities. Additional phenotypes observed in our patients with *DOCK8* duplications include hearing impairment and attention-deficit hyperactivity disorder (ADHD), which was also reported previously by Glessner et al. (2017). Our patients also exhibited undescended testes (2) and atrophy of one testis (1).

**Table 3:** Clinical characterization of the patients with *DOCK8* gains.

Resource GRCh37/hg19 interval of assessed variants	ISCA 52389–416351	Decipher 41587–489842	Krgovic 204193–271316	*N*	%	Our patients		
						D714/10	D684/13	D1748/15
						1–271132	203861–398865	271533–440683
Number of assessed cases	37	19	2	58	100.0			
DD	8	4	1	13	22.41	+	−	−
ASD*	3	6	1	10	17.24	+	+	+
ID	1	6	1	8	13.79	+	−	−
Behavioral abn.	0	4	2	6	10.34	−	−	−
Dysmorphic	3	3	1	7	12.06	+	−	−
Speech delay/disorder	0	2	2	4	6.9	+	+	+
Seizures	2	2	0	4	6.9	−	−	−
Ambiguous genitalia	1	1	0	2	3.45	−	−	−
Obesity	0	2	0	2	3.45	−	−	+
Sleep disturbance	0	1	0	1	1.72	−	−	+
Microcephaly	0	0	1	1	1.72	−	−	−
Dolichocephaly	0	1	0	1	1.72	−	−	−
Plagiocephaly	0	1	0	1	1.72	−	−	−
Craniosynostosis	0	1	0	1	1.72	−	−	−
Cardiac abnorm.	1	0	0	1	1.72	−	−	−
Short stature	0	1	0	1	1.72	−	−	−
Agenesis CC	1	0	0	1	1.72	−	−	−
Feeding problems	0	1	0	1	1.72	−	−	−
Undescended testes	0	0	0	0	0	+	−	+
ADHD*	0	0	0	0	0	+	+	+
Hypacusis	0	0	0	0	0	−	+	+

**Notes:**

CC, corpus callosum.

* Glessner et al. (2017).

Ten CNVs with uncertain significance were found in the cohort (Table 2C). This category included variants that lack entries in the DGV but contained brain-expressed and/or dosage-sensitive genes, such as dup11q14-q21, which spans melatonin receptor 1B and fat tumor suppressor 3 (*FAT3*); del 6q22, which spans protein-tyrosine phosphatase, receptor-type, kappa; dup 17q21.31, which spans granulin and run domain-containing protein 3A; and dup 9q33.2, which spans stomatin and gelsolin. The category also included variants with entries in the DGV that span brain-expressed dosage-sensitive genes for which there is little or no evidence of involvement in the etiology of autism. CNVs in this latter group were del 2p12, which spans catenin alpha-2 (*CTNNA2*); dup 16q11.2, which spans vacuolar protein sorting 35 (*VSP35*); and dup Xp11.22, which spans (*SMC1A*) and 17-beta-hydroxysteroid dehydrogenase X (*HSD17B10*). Other CNVs in this group included variants overlapping OMIM morbid genes associated with etiologies for non-neuropsychiatric disorders, namely dup 5q32.3, del 2q13, and dup 16p12.2, spanning muscle segment homeobox 2, nephrocystin 1, and otoancorin, respectively.

Three patients exhibited multiple CNVs designated as pathogenic, likely pathogenic, or VOUS—uncertain: D1377/15, D1190/16 and D1094/16 (Tables 2B and 2C).

Interestingly, secondary findings, that is, CNVs encompassing genes of interest (here defined as genes associated with autism or expressed in the brain), were discovered in the patient with the *FMR1* mutation. One of these CNVs encompassed the genes acetylserotonin O-methyltransferase (*ASMT*), and the other encompassed *HSD17B10* and *SMC1A*. A paternally inherited deletion/SNV involving the *MAPK3* gene (16p11.2 region) detected by MLPA was also identified in this patient.

Copy number variant encompassing genes involved in the pathophysiology of parkinsonism were detected in three (3.26%) patients. A deletion (covering exons 2–3) and a duplication (covering exon 2) of the gene *PARK2* (6q26) were discovered in two unrelated patients, and duplication of 16q11.2 including the gene *VSP35* (*PARK17*) was discovered in the third.

## Discussion

The detection rate for pathogenic CNV in ASD patients achieved using MLPA was identical to that achieved using CMA. We therefore suggest that MLPA is sufficient to diagnose unambiguously pathogenic variants under some circumstances—for instance, when CMA is unavailable or in cases where the need to interpret VOUS variants or incidental findings would be problematic and patients would be unwilling to deal with the implications of such variants being detected (especially in cases where a prenatal genetic diagnosis would have to be followed up with further investigations within the family). The patients would retain the ability to refuse to be informed of such findings despite their possible pathogenic impact; in such cases, CMA would be redundant because we have confirmed that clinically significant variants can be detected by both methods. This example demonstrates that the benefits of CMA in clinical applications differ from those in research contexts, and shows that CNV analysis by CMA in individuals with ASD can enable diagnosis and appropriate genetic counseling in a small number of cases. CMA is unavailable to some laboratories because it requires specialized equipment. Therefore, many labs would have to make a large capital outlay to perform CMA but will be readily able to perform MLPA with existing equipment to determine the causes of known syndromes. These laboratories can rely on targeted methods such as MLPA to exclude possible causes of syndromic autism. However, MLPA would not be sufficient to detect CNV associated with nonsyndromic ASD. Therefore, CMA remains an invaluable tool for studying the causes of ASD. Our results confirm that CMA outperforms MLPA at detecting CNV classified as VOUS—likely pathogenic. These variants can help reveal new genes involved in the emergence of ASD. Most ASD patients with detected pathogenic CNV are classified as having syndromic autism because the diagnosis of ASD is usually secondary to DD or ID with further comorbidities such as dysmorphic features or growth delay that may suggest the involvement of a syndrome based on the procedures adopted here (see Supplemental Files). Although all the patients in the studied cohort had been assessed by a genetic counsellor before the study was conducted, none of them had been suggested to have any syndrome prior to our testing. This could be partly due to the phenotypic variability of some syndromes.

Even in the boy with BMD, the diagnosis of the syndrome was based on MLPA testing and subsequent confirmation by targeted DNA analysis at 12 years of age. However, the primary reasons given when referring this patient for genetic investigation were severe growth delay, dystrophia together with autistic features. Because the patient’s dystrophia was milder than in DMD, the case was classified as BMD. The typical VCFS phenotype did not manifest in the patient with microdeletion 22q11.2 because the deleted interval did not include the *TBX1* or *HIRA* genes—the deletion was rather distal, spanning the ASD candidate gene *LZTR1* (Krumm et al., 2015). The distal microdeletion 22q11.2 has been linked to behavioral and psychiatric impairments (Burnside, 2015). In the patient with ring chromosome 22, MLPA confirmed the suspected loss of the terminal part of chromosome 22 and the loss of the *SHANK3* gene, which has been associated with ASD (Durand et al., 2007; Nemirovsky et al., 2015). These findings explained the patient’s phenotype and resulted in a diagnosis of Phelan–McDermid syndrome. The most notable aspects of the patient’s phenotype were severe DD and neuropsychiatric impairment (recently described as low functioning autism). However, at the age (24 months) when the girl was tested, the syndrome’s hallmarks had not fully manifested. Further testing was required in four patients with CNV VOUS—likely pathogenic and six patients with CNV VOUS—likely benign or benign because there may have been pathogenic CNV outside the loci covered by the tested probemixes in these cases. The detection rate of MLPA depends somewhat on the chosen probemix, but is comparable to that for CMA if one restricts one’s focus to clinically well described recurrent pathogenic CNV. This makes MLPA a convenient method for fast, reliable, and inexpensive targeted exclusion of CNV involved mostly in syndromic autism. The detection rate for pathogenic or likely pathogenic CNV by CMA in ASD patients ranges from 3 to 30% depending on the cohort and acceptance criteria (Nava et al., 2013; Wang et al., 2016; Guo et al., 2017; Shen et al., 2010; Bremer et al., 2011; Cappuccio et al., 2016; Leppa et al., 2016). In this work, CMA revealed eight index cases with pathogenic CNV. This result is comparable to previous reports (Nava et al., 2013; Shen et al., 2010; Bremer et al., 2011). CMA achieved a higher detection rate for potentially pathogenic variants than MLPA or karyotyping in this work. However, without karyotyping it would have been very difficult to determine the mechanism of occurrence of deletion 22q13.33 (which was due to ring 22). Nor would we have detected the balanced chromosomal rearrangements in two patients. The possibility that these rearrangements may have contributed to the etiology of ASD in these patients cannot be completely excluded.

We observed a relatively high frequency of CNV encompassing genes associated with parkinsonism in our group of ASD patients. Variants encompassing *PARK2* have previously been detected in ASD patients (Nava et al., 2013; Yin et al., 2016). However, we also detected a CNV involving *VSP35* (*PARK17*) in one of our patients whose genome contained multiple CNV. This gene has been suggested to play a role in parkinsonism (Zimprich et al., 2011). The relatively high frequency of CNV overlapping genes associated with this disease raises the possibility that these patients may have an elevated risk of developing parkinsonism in adulthood. An increased frequency of parkinsonism among ASD patients has previously been reported (Starkstein et al., 2015). However, the role of parkinsonism-related genes in the etiology of ASD is currently unclear. It is possible that different kinds of genomic changes affecting certain genes can lead to different phenotypes (Scheuerle & Wilson, 2011).

Two patients exhibited variants encompassing two melatonin-related genes: *ASMT* and the *MNTR1B*. Both genes have been identified as potentially affecting the risk of ASD (Cai et al., 2008; Nava et al., 2013; Jonsson et al., 2010; Anderson et al., 2009). The duplication region 11q14.3-q21 encompassing *MNTR1B* and *FAT3* co-occurred with duplication Xq27.3 encompassing the *SOX3* gene, which was previously linked to the etiology of ID, hypopituitarism, and speech disorders, but not ASD (Solomon, 2004; Stankiewicz et al., 2005).

Two patients exhibited variants overlapping genes encoding catenins (cadherin-associated proteins): deletion 10q21.3 overlaps catenin alpha 3 (*CTNNA3*), and deletion 2p12 overlaps *CTNNA2*. The first *CTNNA3* deletion co-occurred with other CNV—deletion 8p23.2 (*CSMD1*), duplication 16q11.2 (*VSP35*), and duplication 1q42.2 (*DISC1*). While variations in *CTNNA3* have been described in patients with ASD, variations in *CTNNA2* have not (Folmsbee et al., 2016; Bacchelli et al., 2014). *CTNNA2* is predominantly expressed in the brain and helps regulate the stability of synaptic contacts and axogenesis, brain morphogenesis, dendrite morphogenesis, and synapse structural plasticity (The UniProt Consortium, 2017), making it a plausible candidate for involvement in the etiology of ASD.

Duplications covering the gene *DOCK8* were identified in multiple patients by both MLPA and CMA. *DOCK8* encodes a member of the Dock protein family of atypical Rho guanine nucleotide exchange factors for Rac and/or Cdc42 GTPases that play pivotal roles in various processes of brain development. To date, 11 members of the Dock family have been identified in mammals. Dock proteins regulate the actin cytoskeleton, cell adhesion, and dendritic migration (Gadea & Blangy, 2014). There is also evidence that members of the Dock family are associated with several neurodegenerative and neuropsychiatric diseases, including Alzheimer’s disease and ASD (Shi, 2014). Homozygous loss of function of the *DOCK8* gene causes autosomal recessive hyper-IgE recurrent infection syndrome (Zhang et al., 2009). In addition, evidence was recently presented supporting a causal relationship between heterozygous disruption of *DOCK8* and mental retardation, pervasive developmental disorders, autism, and bipolar disorders (Nava et al., 2013; Wang et al., 2016; Griggs et al., 2008; Glessner et al., 2017; Krgovic et al., 2018). Our results support the findings of Glessner et al. (2017), who identified *DOCK8/KANK1* as novel significant loci for ASD and ADHD. We observed significant enrichment of CNV involving gains of the *DOCK8* gene in the studied ASD cohort. This may indicate that the region of the *DOCK8/KANK1* locus associated with ASD and ADHD is likely to be within or proximal to the gene *DOCK8*. Our patients with *DOCK8* gains had all been diagnosed with ADHD. We detected no individuals with any CNV overlapping with the *DOCK8* gene in our control cohort, so we regard this gene as an interesting candidate for further study on the etiology of ASD. Because the *DOCK8* duplication was inherited from a healthy father in one case, we assume that variant increases the risk of ASD or other neuropsychiatric conditions but that its phenotypic impact may be limited by incomplete penetrance or/and variable expressivity. Both of these factors are known to complicate genetic counseling in patients with CNV encompassing neurosusceptibility loci.

**Figure 2:**
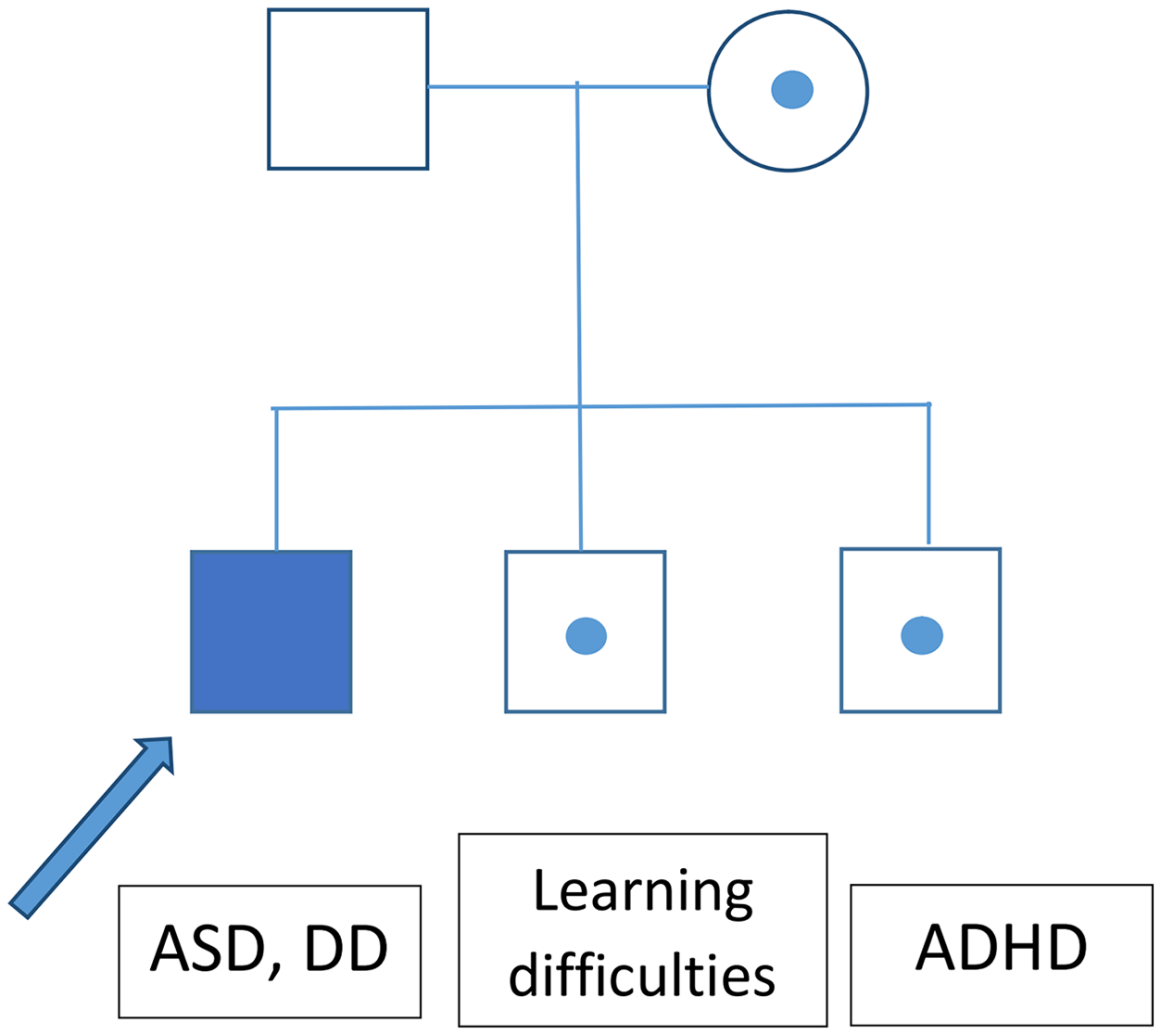
Segregation analysis for the family of a patient bearing a CNV causing loss of *NRXN1*.

The greatest limitation of this study, aside from the relatively small cohort, is the lack of information about the inheritance of most of the identified CNV. It seems that CNVs with variable expressivity (del/dup16p11.2, *NRXN1*) are frequently inherited. An analysis of ASD patients’ parental genomes could thus help to explain the patients’ phenotypes. A segregational analysis was performed for the family with a heterozygotic loss in *NRXN1*, revealing that this CNV exhibited incomplete penetrance (Fig. 2).

## Conclusion

Diagnosis was achieved in only eight index cases (8.7% of the studied cohort), all of which involved patients classified as having syndromic autism. The rate of pathogenic CNV detection by CMA was identical to that achieved with MLPA using probemixes targeted toward losses associated with ID, DD, and ASD. Consequently, our results indicate that MLPA and CMA are equally reliable methods for obtaining clinically relevant findings. Therefore, MLPA can be used as a quick alternative to CMA for excluding syndromes associated with ASD. MLPA is also frequently used to confirm CMA findings, and for targeted verification of the origins of CNV during parental testing. However, many genes are involved in the etiology of ASD, creating a clear need for whole-genome screening to identify genes associated with ASD and to clarify the clinical impact of VOUS. Moreover, increasing knowledge of knew candidate genes in ASD provided by CMA (or NGS) enables to develop the new targeted tests (MLPA probemixes, targeted arrays) for quick exclusion known pathogenic mutations in particular patients. Our results confirm that traditional karyotyping is an indispensable tool for deciphering the origins of specific CNV and detecting balanced chromosomal changes, the clinical significance of which cannot be totally disregarded in ASD patients. This work presents further evidence that genes identified in the etiology of various genetic conditions can be linked to the pathophysiology of ASD (*DOCK8*). However, the exact pathophysiological mechanism underlying the functions of these genes in the development of phenotypes such as ASD remains unknown.

## Supplemental Information

10.7717/peerj.6183/supp-1 Supplemental Information 1 Phenotype of the ASD patients. *hearing impairment.
**the patient was born from IVF.
ID–intellectual disability, DD–developmental delay, ADHD–attention hyperactivity disorder, NA–not available, M–male, F–female.

## Abbreviations

ASD: autism spectrum disorders
PDD-NOS: pervasive developmental disorder-not otherwise specified
MLPA: multiplex ligation-dependent probe amplification
CMA: chromosomal microarray analysis
CNVs: copy number variants
VOUS: variant of unknown significance
ID: intellectual disability
DD: developmental delay.

## Additional Information and Declarations

### Microarray Data Deposition

The following information was supplied regarding the deposition of microarray data: The data discussed in this publication have been deposited in NCBI’s Gene Expression Omnibus database. Accession number: GSE114870.

### Data Availability

The following information was supplied regarding data availability: Srovnal, Josef (2018): ASD data. figshare. Dataset. DOI 10.6084/m9.figshare.6236648.v1.
